# Early detection of chronic obstructive pulmonary disease in primary care: a randomised controlled trial

**DOI:** 10.3399/BJGP.2022.0565

**Published:** 2023-10-31

**Authors:** Anthony Chapron, Emilie Andres, Laure Fiquet, Fabienne Pelé, Emmanuel Allory, Estelle Le Pabic, Aurélie Veislinger, Lisa Le Guillou, Stéphanie Guillot, Bruno Laviolle, Stéphane Jouneau

**Affiliations:** Department of General Practice, University of Rennes, Centre Hospitalier Universitaire (CHU) Rennes, Rennes; UMR_S 1085, Institut National de la Santé et de la Recherche Médicale (INSERM), Institut de Recherche en Santé, Environnement et Travail (IRSET), Rennes.; Department of General Practice, University of Rennes, CHU Rennes, Rennes; CIC-1414, INSERM, Rennes.; Department of General Practice, University of Rennes, CHU Rennes, Rennes; CIC-1414, INSERM, Rennes.; CIC-1414, INSERM, Rennes.; Department of General Practice, University of Rennes, CHU Rennes, Rennes; CIC-1414, INSERM, Rennes.; CIC-1414, INSERM, Rennes.; Department of General Practice, University of Rennes, CHU Rennes, Rennes; CIC-1414, INSERM, Rennes.; Department of Respiratory Medicine, University of Rennes, CHU Rennes, Rennes.; Department of Respiratory Medicine, University of Rennes, CHU Rennes, Rennes.; CIC-1414, INSERM, Rennes; UMR_S 1085, INSERM, IRSET, Rennes.; UMR_S 1085, INSERM, IRSET, Rennes; Department of Respiratory Medicine, University of Rennes, CHU Rennes, Rennes.

**Keywords:** chronic obstructive pulmonary disease, early detection, general practice, GOLD questions, primary care

## Abstract

**Background:**

Worldwide, chronic obstructive pulmonary disease (COPD) remains largely underdiagnosed.

**Aim:**

To assess whether the use of Global Initiative for Chronic Obstructive Lung Disease (GOLD) questions and COPD coordination, either alone or combined, would detect new COPD cases in primary care.

**Design and setting:**

GPs in Brittany, France, systematically enrolled patients aged 40–80 years over a 4-month period in this French multicentre cluster randomised controlled study.

**Method:**

GPs were randomly allocated to one of four groups: control (standard of care), GOLD questions (adapted from symptoms and risk factors identified by GOLD), COPD coordination, and GOLD questions with COPD coordination. New cases of COPD were those confirmed by spirometry: post-bronchodilator forced expiratory volume in 1 second over forced vital capacity of <0.7.

**Results:**

In total, 11 430 consultations were conducted by 47 GPs, who enrolled 3162 patients who did not have prior diagnosed asthma or COPD. Among these, 802 (25%) were enrolled in the control, 820 (26%) in the GOLD questions, 802 (25%) in the COPD coordination, and 738 (23%) in the GOLD questions with COPD coordination groups. In the control group, COPD was not evoked, and no spirometry was prescribed. All new cases of COPD diagnosed (*n* = 24, 0.8%) were in the intervention groups, representing 6.8% of patients who performed spirometry. Statistically significantly more new cases of COPD were detected with COPD coordination (*P* = 0.01).

**Conclusion:**

Interventions that can be easily implemented, such as the GOLD questions and COPD coordination, can identify new cases of COPD. Studies are needed to identify the most appropriate case-finding strategies for GPs to detect COPD in primary care for each country.

## INTRODUCTION

Chronic obstructive pulmonary disease (COPD) is a leading cause of morbidity and mortality worldwide.^1^ However, COPD remains largely underdiagnosed.^2^^–^5 An early detection strategy that actively identifies patients with risk factors or early symptoms of COPD would allow for the earlier detection of patients with COPD. Early detection, during COPD evolution, has been shown to control COPD evolution and symptoms.^6^^–^8

The leading risk factor for COPD is a history of smoking, either current or past.^9^ Patients who are symptomatic and have suspected COPD require spirometry to confirm the COPD diagnosis.^10^ Various screening tools and disease- specific questionnaires have been used in primary care to identify patients at risk of COPD.^5^^,^^11^

Most COPD is detected and treated in primary care, but diagnosis is frequently delayed and often only occurs after COPD exacerbation.^9^ As primary care physicians underuse spirometry to confirm COPD diagnosis,^12^^,^^13^ numerous patients with suspected COPD remain without a confirmed diagnosis.^13^^–^15 In patients who have not had spirometry, GPs tend to underestimate COPD diagnosis or severity and, therefore, patients may not receive early and/or optimal treatment.^12^

Current guidelines recommend that COPD diagnosis should be considered in patients who have symptoms and/or are at risk of COPD;^10^^,^^16^ as such, systematic COPD screening is not recommended in primary care. In French primary care, approximately 7.5% of patients aged >40 years are expected to have COPD.^17^ Targeted screening to identify patients with risk factors and early symptoms is recommended.

Symptoms and risk factors have been identified in the annual Global Initiative for Chronic Obstructive Lung Disease (GOLD) reports as clinical indicators for considering a diagnosis of COPD.^9^^,^^18^ Haute Autorité de Santé (HAS), France’s national health authority, recommended that these be adapted into French in the form of questions (hereafter referred to as ‘GOLD questions’).^19^ These seemed to be useful and practical for COPD screening in primary care, which is the view of GPs in practice and the HAS recommendation.^19^

**Table:** How this fits in

In primary care, the use of questions adapted from symptoms and risk factors identified by the Global Initiative for Chronic Obstructive Lung Disease (GOLD) and chronic obstructive pulmonary disease (COPD) coordination to facilitate spirometry access, either alone or combined, facilitates COPD detection. These interventions are relatively easy to implement in everyday clinical practice and can be adapted for countries in which most GPs are not trained to perform spirometry.

In France, most GPs working in the private sector do not perform spirometry. When COPD is suspected, patients are referred to specialists, mainly pulmonologists. The pulmonologist diagnoses and initiates treatment but, after diagnosis, it is most frequently GPs who manage patients who have COPD.

There is a growing need to detect COPD early, when preventive and therapeutic interventions are expected to be most effective.^2^ As a result, the Détection Précoce en Soins Primaires de la BPCO (DISCO) study was designed by the authors to assess two interventions to detect patients with COPD in primary care — namely, the use of the GOLD questions and the use of COPD coordination (in a coordinated COPD pathway). These interventions were assessed alone and combined, compared with standard care.

## METHOD

**Figure 1.**
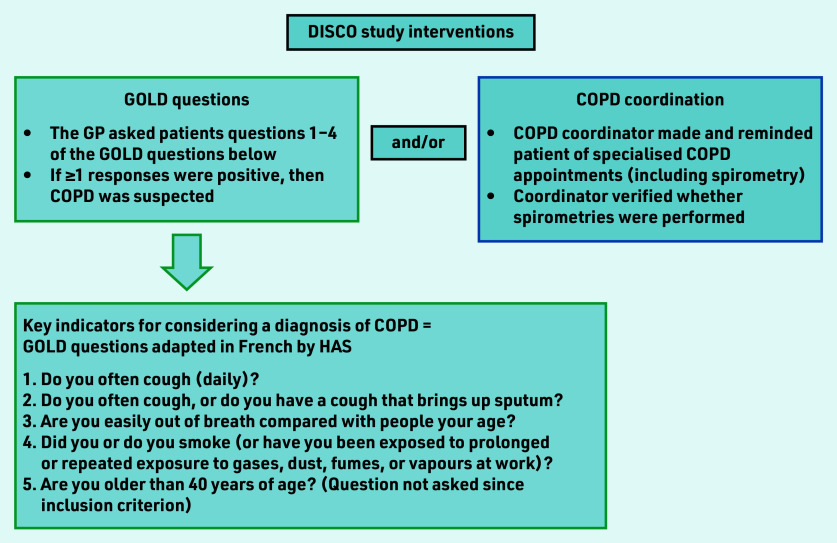
*Study interventions for detecting COPD assessed in the DISCO study.^9^^,^^20^* *COPD = chronic obstructive pulmonary disease. DISCO = Détection Précoce en Soins Primaires de la BPCO. GOLD = Global Initiative for Chronic Obstructive Lung Disease. HAS = Haute Autorité de Santé (French National Authority for Health).*

DISCO was a multicentre, clustered, randomised controlled study; Chapron *et al*^20^ details the results of the DISCO pilot study. A 2 × 2 factorial design assessed two healthcare interventions — GOLD questions and COPD coordination — alone or combined, for detecting COPD in primary care (Figure 1).

Among the five GOLD questions (Figure 1), three concern COPD symptoms, and one concerns smoking and/or exposure to other airborne gases and particles. The GPs who were allocated the GOLD questions asked their patients every question, except that concerning age — this is because being aged >40 years was an inclusion criterion of the study. A positive response to any question was sufficient to suspect COPD and prescribe spirometry.

**Figure 2.**
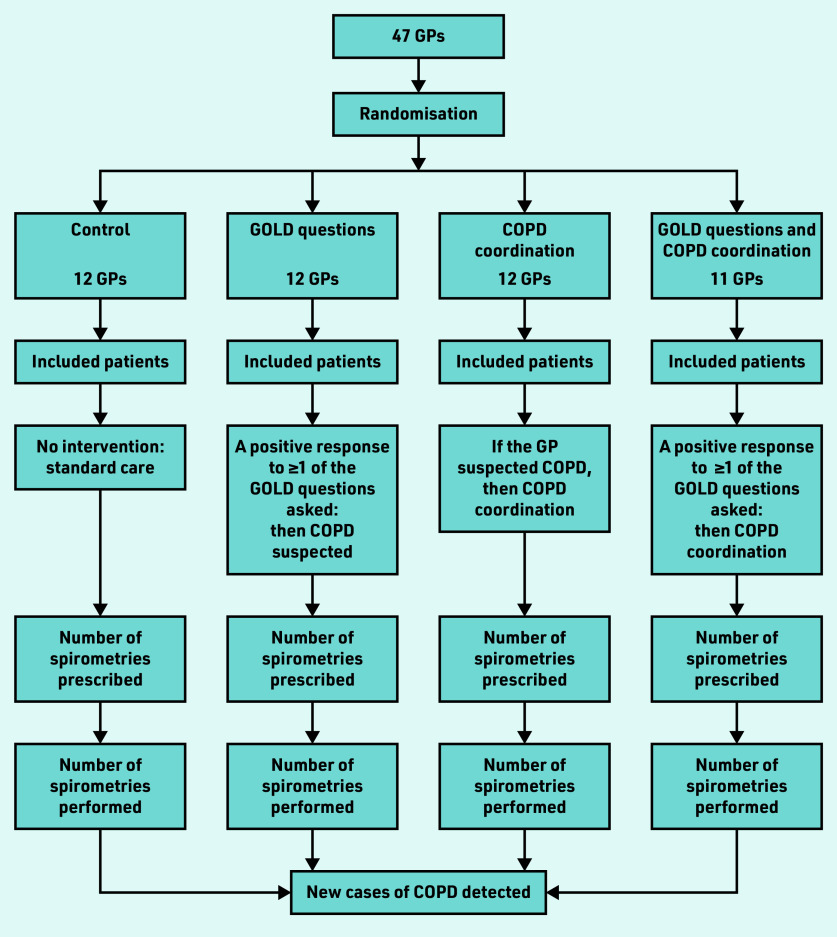
*Study design.* *COPD = chronic obstructive pulmonary disease. GOLD = Global Initiative for Chronic Obstructive Lung Disease.*

GPs who were assigned to COPD coordination alerted the dedicated COPD coordinator when COPD was suspected. Once alerted, the coordinator organised and facilitated the specialised COPD consultations. The coordinator performed a role similar to that of a medical assistant — that is, making appointments with the pulmonologist or the specialist performing spirometry, reminding patients of their appointments, rescheduling them if required, and confirming that spirometry had been performed. The coordinator also managed the patients of all investigating practices, whether they were allocated to the COPD coordination group or the GOLD question and COPD coordination group. The study design is illustrated in Figure 2.

### GPs and randomisation

GPs in Brittany, France, in private or group practices, were solicited for this study by the Department of General Practice, University of Rennes. Initially, GPs knew that the study concerned early detection of chronic diseases, but not specifically COPD; this was done to maintain blinding for GPs and patients in the control group. GPs were cluster randomised to avoid contamination bias: a GP corresponded to a cluster and GPs from the same practice were assigned to the same cluster. Software was used to automatically randomise the GP clusters to one of four study groups: control, GOLD questions, COPD coordination, and GOLD questions with COPD coordination groups.

In the control group (standard care), GPs received no information about disease and study interventions, and patient eligibility was retrospectively reviewed by a clinical research associate using medical records. The GPs in the three intervention groups were trained independently according to their specific study group. Among the 350 GPs from the network of the Department of General Practice, University of Rennes, 47 GPs from 45 practices participated: 12 GPs were allocated to the control group, 12 to the GOLD question group, 12 to the COPD coordination group, and 11 to the GOLD questions and COPD coordination group.

### Patients

Patients were eligible to participate in the study if, during the 4-month enrolment period, they were:

- aged 40–80 years;
- benefitting from the social security system, that is, covered by health insurance;
- willing to participate; and
- consulting for any matter on the day chosen for inclusion at the centre.

Patients were excluded from participating if they:

- had confirmed COPD or confirmed asthma;
- were unable to perform spirometry;
- were pregnant; and
- were patients under guardianship.

Patients underwent the COPD screening strategy allocated to their GP.

### Data collection

The following patient data were collected:

- demographics;
- medical history;
- history of smoking;
- occupation;
- environmental and occupational risk factors; and
- habitus.

In patients sent for specialised COPD consultations and spirometry, the consultation reports were collected. The forced expiratory volume in 1 second (FEV_1_)/forced vital capacity (FVC) ratio before and after reversibility testing, as well as the time intervals between GP consultations and the spirometry, were collected.

### Study outcomes

The primary objective was to determine whether the interventions (the GOLD questions and/or the COPD coordination) would detect more new cases of COPD in primary care. Patients with an FEV_1_/FVC ratio of <0.7, after bronchodilation, were considered to be diagnosed with COPD. The primary outcome, in each group, was the proportion of new cases of COPD detected relative to the number of patients participating. Two independent pulmonologists performed a blinded centralised review of all spirometry reports; in cases of discordance, a third review by a third pulmonologist determined the outcome. Patients not performing a spirometry within 6 months of the request for consultation were considered not diagnosed with COPD.

Secondary outcomes comprised the description of the results of specialised consultations with spirometry and the description of patients with new cases of COPD.

### Statistical analysis

To assess the difference between detecting COPD in the study groups, with a power of 95%, an alpha risk of 2.5%, and considering the cluster randomisation of the medical practices (with a coefficient intra-cluster of 0.01),^15^ it was estimated that 3040 patients in 32 medical practices were required. This sample size was based on an estimated 1% COPD prevalence in the control group,^16^^,^^17^^,^^20^ 2% in the GOLD questions group, 2% in the COPD coordination group,^18^^,^^19^ and 5% in the GOLD questions and COPD coordination group.^21^^,^^22^

Quantitative variables were given as a median with interquartile range (IQR) and compared using Mann–Whitney tests. Qualitative variables were given as numbers with percentages, and compared using χ^2^ or Fisher exact tests. The effects of the interventions, GOLD questions, and/or COPD coordination on the number of new cases of COPD detected were estimated using a step-by-step logistical regression model. A post-hoc analysis assessed whether COPD coordination shortened the time interval to spirometry. Statistical significance was set at *P*<0.05. Missing data were not replaced. Statistical analyses were performed using SAS (version 9.4).

### Ethics

The DISCO study was performed in line with French data-protection laws and ethic committee. It was approved by the Commission Nationale de l’Informatique et des Libertés and conformed to the Reference Methodology MR-003. All patients gave oral non-opposition for the study. DISCO was registered in ClinicalTrials.gov (reference: NCT03046199).

## RESULTS

**Figure 3.**
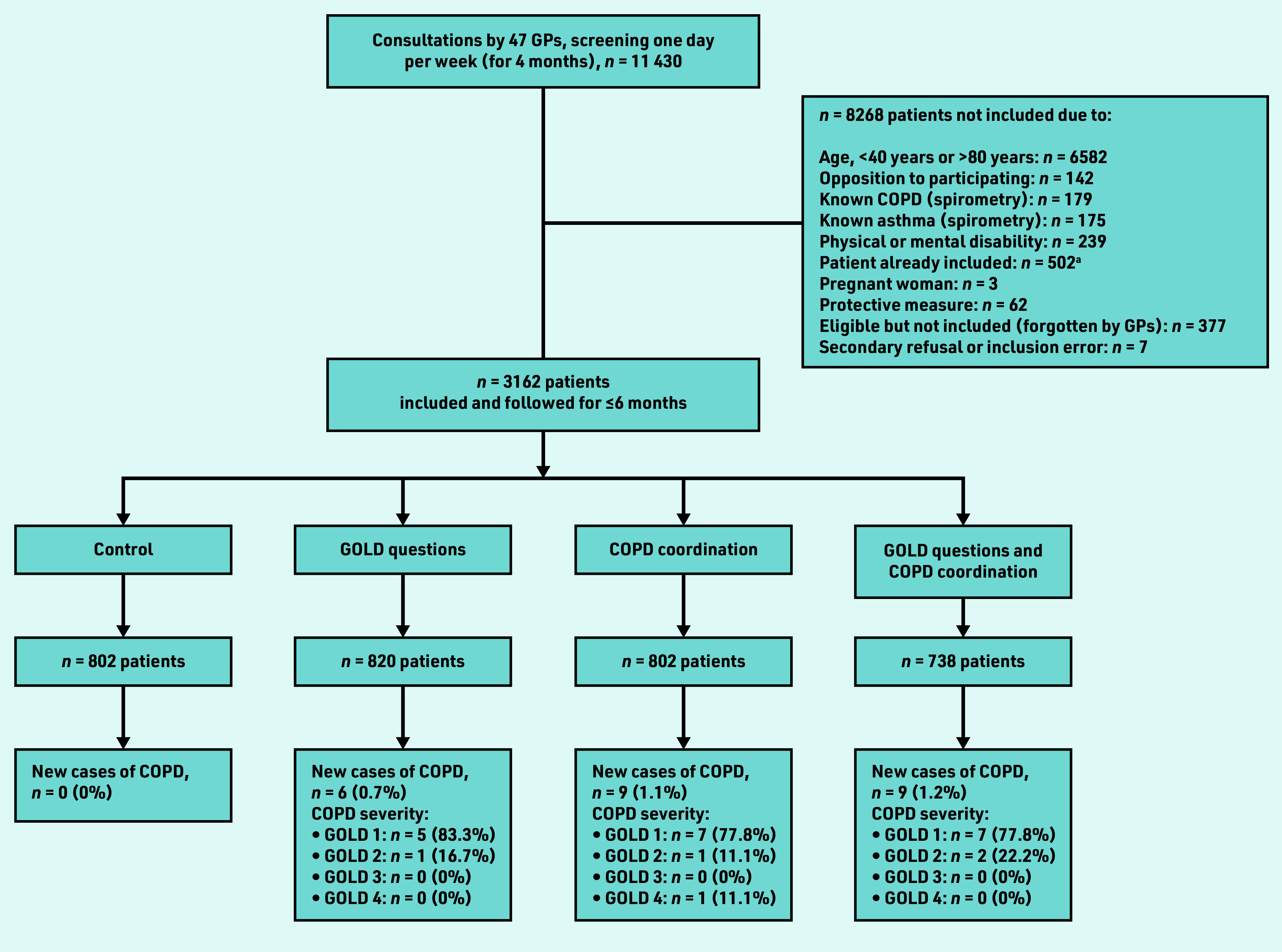
*Diagnosis of new cases of COPD according to the group allocated.* *a One patient could be seen more than once; 502 patients were thus excluded. COPD = chronic obstructive pulmonary disease. GOLD = Global Initiative for Chronic Obstructive Lung Disease.*

During the 4-month enrolment period (1 October 2018 to 31 January 2019), 11 430 patients consulted 47 GPs for any matter. Of these, 3162 patients were aged 40–80 years, not diagnosed with asthma or COPD, and allocated to the control group (*n* = 802, 25%), GOLD questions (*n* = 820, 26%), COPD coordination (*n* = 802, 25%), and GOLD questions with COPD coordination (*n* = 738, 23%) (Figure 3).

The results presented here show that a COPD coordinator facilitated spirometry and statistically significantly increased the detection of new COPD cases. Using COPD coordination tended to shorten the time interval to spirometry, but this was not statistically significant. For patients in the COPD coordination only group, a lower proportion of spirometry tests were prescribed, but a greater proportion were actually performed.

Follow up to ensure spirometry was performed was one of the roles of the coordinator.

### Patient characteristics

The 3162 patients enrolled had a median age of 59 years (IQR 49–68) and 54% were female. The baseline characteristics of the patients enrolled in the study groups were similar for sex, body mass index, and smoking status (Table 1).

**Table 1.** Patient baseline characteristics

**Patient characteristics**	**Overall**	**Control group**	**GOLD questions**	**COPD coordination**	**GOLD questions** **and coordination**	***P*-value**
**Total,** ***n***	3162	802	820	802	738	

**Median age, years (IQR)**	59 (49–68)	60 (49–69)	60 (50–69)	60 (51–69)	57 (48–66)	<0.001

**Sex,** ***n* (%)**						0.06
Female	1713 (54)	418 (52)	468 (57)	448 (56)	379 (51)	
Male	1449 (46)	384 (48)	352 (43)	354 (44)	359 (49)	

**Median BMI, kg/m^2^ (IQR)**	26.2 (23.4–29.8)	26.1 (23.3–29.8)	26.7 (23.5–30.5)	26.0 (23.5–29.4)	26.2 (23.3–29.4)	0.25
Missing data, *n* (%)	572 (18)	67 (8)	319 (39)	37 (5)	149 (20)	

**Smoking status,** ***n* (%)**						<0.001
Never smoker	1637 (52)	440 (55)	451 (55)	429 (53)	317 (43)	
Former smoker	881 (28)	182 (23)	233 (28)	212 (26)	254 (34)	
Current smoker	572 (18)	132 (16)	134 (16)	139 (17)	167 (23)	
Missing data	72 (2)	48 (6)	2 (0.2)	22 (3)	0 (0)	
Median number of pack-years, *n* (IQR)	19 (12–30)	25 (15–30)	15 (10–30)	25 (20–39)	17 (10–30)	0.42
Median smoking duration in years for former and current smokers, *n* (IQR)	24 (15–60)	25 (17–55)	25 (15–60)	22 (15–54)	22 (14–50)	0.25

**With occupational risk factors,** ***n* (%)^a^**	475 (15)	76 (9)	144 (18)	125 (16)	130 (18)	0.001
Missing data	208 (7)	118 (15)	0 (0)	90 (11)	0 (0)	

**Patients with addictions,** ***n* (%)**	198 (6)	59 (7)	39 (5)	37 (5)	63 (9)	0.002
Missing data	70 (2)	29 (4)	0 (0)	40 (5)	1 (0.1)	

**Addictions,** ***n* (%)^b^**	210	60	39	38	73	
Alcohol	177 (84)	52 (87)	37 (95)	35 (92)	53 (73)	0.27
Cannabis	15 (7)	1 (2)	1 (3)	2 (5)	11 (15)	0.01
Other narcotic drugs	7 (3)	1 (2)	0 (0)	0 (0)	6 (8)	0.03
Medication	11 (5)	6 (10)	1 (3)	1 (3)	3 (4)	0.42

a *Risk factors established by the Haute Autorité de Santé (French National Authority for Health): mining sector (exposure to silica, works at the bottom of coal or iron mines, and/or inhalation of iron oxide dusts or fumes); building and public works (tunnelling, paving of roads, and other construction works with chronic exposure and/or excessive levels of gas/dust/vapours); foundry and steel industry (exposure to several mineral particles [metal dusts, coal, and silica], exposure to gases or fumes [furnace emissions, metal fumes, sulphur, and nitrogen oxide]); textile industry (spinning mill workers in, for example, cotton, linen, hemp, and sisal); and agricultural occupations (occupations concerned with the use of pesticide-type products, grain industry [elevator workers, dock workers, and milling employees], milk production, and pig and poultry farming).*

b *Multiple responses were possible. BMI = body mass index. COPD = chronic obstructive pulmonary disease. GOLD = Global Initiative for Chronic Obstructive Lung Disease. IQR = interquartile range.*

### Newly detected cases of COPD

Among the 3162 patients included, 827 (26%) patients had a risk factor, early symptom, or clinical presentation suggesting COPD, and were prescribed spirometry (Table 2). In the control group, COPD was not evoked, and no spirometry was prescribed for any of the 802 patients. In total, 24 (0.8%) new cases of COPD were diagnosed, all of which involved patients in the intervention groups, representing 6.8% of patients who performed spirometry. The study did not show an additive effect of the two interventions (GOLD and COPD coordination, *P* = 0.11); however, statistically significantly more new COPD cases were detected with COPD coordination (*P* = 0.01).

**Table 2.** Spirometry examinations prescribed and performed, time interval to spirometry, and new cases of COPD diagnosed

**Category**	**Overall**	**Control group**	**GOLD questions**	**COPD coordination**	**GOLD questions and coordination**
**Total, *n***	3162	802	820	802	738

**Spirometry indicated by GOLD questions, *n* (%)**	—	—	470 (57)	—	492 (67)
Spirometry prescribed	827 (26)	0 (0)	309 (38)	153 (19)	365 (49)
Spirometry performed	351 (42)	0 (0)	102 (33)	76 (50)	173 (47)
Spirometry not performed	476 (58)	0 (0)	207 (67)	77 (50)	192 (53)

**Reason why spirometry was not performed, *n* (%)**					
Lack of time	23 (5)	n/a	2 (1)	8 (10)	13 (7)
Appointment >6 months after GP consultation	5 (1)	n/a	4 (2)	0 (0)	1 (1)
Accessibility problem (lack of transport)	4 (1)	n/a	0 (0)	0 (0)	4 (2)
Accessibility problem (distance)	0 (0)	n/a	0 (0)	0 (0)	0 (0)
Patient refusal	48 (10)	n/a	25 (12)	9 (12)	14 (7)
Patient health	22 (5)	n/a	14 (7)	7 (9)	1 (1)
Personal reason	4 (1)	n/a	3 (1)	1 (1)	0 (0)
Unknown	370 (78)	n/a	159 (77)	52 (68)	159 (83)

**Median time interval, months between GP consultation and spirometry, *n* (IQR)**	2.9 (1.5–4.3)	n/a	3.2 (1.0–5.4)	3.0 (2.3–3.9)	2.5 (1.4–4.0)

**New cases of COPD diagnosed, *n* (%)**	24 (0.8)	0 (0.0)	6 (0.7)	9 (1.1)	9 (1.2)

*COPD = chronic obstructive pulmonary disease. GOLD = Global Initiative for Chronic Obstructive Lung Disease. IQR = interquartile range. n/a = not applicable.*

In the 24 patients diagnosed with new cases of COPD, eight (33%) were female and the median age was 63 years (IQR 57–69 years) (Table 3). Most patients with newly detected cases of COPD were either current or former smokers (*n* = 22, 92%, *P*<0.0001). All patients who responded to GOLD questions had a tobacco or professional exposure risk factor. Two (8%) patients with newly detected cases of COPD had only occupational risk factors (data not shown). In current or former smokers, the mean tobacco consumption was 25.3 pack– years (Table 3).

**Table 3.** Characteristics of patients with newly detected cases of COPD

**Patient characteristics**	**Overall**	**GOLD questions**	**COPD coordination**	**GOLD questions and coordination**
**Total, *n***	24	6	9	9

**Median age, years (IQR)**	63 (57–69)	67 (57–71)	63 (60–65)	60 (52–72)

**Sex, *n* (%)**				
Female	8 (33)	2 (33)	5 (56)	1 (11)
Male	16 (67)	4 (67)	4 (44)	8 (89)

**Smoking status, *n* (%)**				
Never smoker	2 (8)	0 (0)	1 (11)	1 (11)
Former/current smoker	22 (92)	6 (100)	8 (89)	8 (89)

**Mean pack–years for former and current smokers, *n* (SD)**	25.3 (17.4)	23.1 (12.2)	37.0 (19.0)	15.1 (10.9)

**Number of positive responses to GOLD questions, *n* (%)**				
Do you often cough (daily)?	6 (25)	2 (33)	Not collected^a^	4 (44)
Do you often cough, or do you have a cough that brings up sputum?	6 (25)	2 (33)	Not collected^a^	4 (44)
Are you easily out of breath compared with people your age?	8 (33)	2 (33)	Not collected^a^	6 (67)
Did you, or do you, smoke (or have you been exposed to prolonged or repeated exposure to gases, dust, fumes, or vapours at work)?	15 (63)	6 (100)	Not collected^a^	9 (100)
GOLD question data not collected^a^	9	0	9	0

**Mean spirometry result, % (SD)**				
FEV_1_/FVC before bronchodilation	63.8 (4.7)	60.6 (5.0)	64.1 (4.1)	65.2 (4.2)
FEV_1_ % predicted before bronchodilation	87.9 (21.3)	79.8 (9.6)	88.3 (27.6)	91.9 (17.2)
FEV_1_/FVC after bronchodilation	63.5 (6.9)	61.5 (5.6)	63.3 (9.5)	65.1 (3.3)
FEV_1_ % predicted after bronchodilation	88.1 (21.1)	77.0 (9.2)	90.1 (27.8)	93.6 (15.7)

a *As the GOLD questions were not used in the COPD coordination group, these data were not collected. COPD = chronic obstructive pulmonary disease. FEV_1_ = forced expiratory volume in 1 second. FVC = forced vital capacity. GOLD = Global Initiative for Chronic Obstructive Lung Disease. IQR = interquartile range. SD = standard deviation.*

Overall, 19 of the 24 (79%) new cases of COPD detected were of GOLD 1 severity, four (17%) GOLD 2 severity, and one (4%) with GOLD 4 severity (Figure 3).

### Specialised consultations with spirometry

Spirometry was prescribed for 827 (26%) of the 3162 patients; none of these were in the control group. The 827 patients for whom spirometry was prescribed comprised 35% of the 2360 patients enrolled in the three intervention groups (Table 2). Overall, spirometry was performed for 351 (42%) of the 827 individuals for whom it had been prescribed: 102 (33%) were in the GOLD questions group, 76 (50%) in the COPD coordination group, and 173 (47%) in the GOLD questions and coordination group. The 351 consultations with spirometry also made it possible to diagnose or evoke 151 other pathologies in 131 (37%) patients, including 14 cases of asthma, 30 cases of non-specific obstructive syndromes, and 25 cases of restrictive syndromes that required further explorations (data not shown). Two patients with pleural plaques were identified in the intervention groups.

The median time interval from consultation until spirometry was 2.9 months (IQR 1.5–4.3 [Table 2], *P* = 0.12). The COPD coordinator intervened in 249 (71%) of the 351 patients for whom spirometry was performed (data not shown). The median time interval to spirometry was 2.7 months (IQR 1.8–3.9) for patients with COPD coordination compared with 3.2 (IQR 1.0– 5.4) for those without COPD coordination (*P* = 0.24) (Table 2).

## DISCUSSION

### Summary

No new cases of COPD were detected in the control group, but these were detected in the intervention groups. The use of COPD coordination statistically significantly increased the detection of new COPD cases in French primary care.

Most studies^5^^,^^11^^,^^21^^–^25 have assessed interventions based on questionnaires to detect COPD. The GOLD questions helped to detect COPD in French primary care, like other questionnaires for identifying underdiagnosed COPD or patients at risk of COPD.^11^^,^^24^ The study groups with GOLD questions detected more new cases of COPD than the control group, but were not superior to the COPD coordination group. These questions provide GPs with a useful tool to actively and opportunistically seek information about COPD, allowing for early detection.^26^ However, GOLD questions did not bring any added value for clinically evoking COPD: statistically significantly more patients with new cases of COPD were detected with the COPD coordination intervention, with or without GOLD questions. Combining COPD coordination with GOLD questions did not statistically significantly increase COPD detection.

### Strengths and limitations

To the authors’ knowledge, DISCO is one of few controlled randomised studies worldwide, and the only one conducted in France, to assess interventions to detect COPD in primary care. In addition, a substantial number of patients were recruited to the study. The investigators failed to include 377 eligible patients, which could have further strengthened the results.

The DISCO study included patients on the basis of occupational risk factors (GOLD questions), which allowed new cases of COPD to be identified in non-smoking patients.

### Comparison with existing literature

Although 11 430 patients were screened and 3162 patients were enrolled, the study detected only 24 (0.8%) new cases of COPD; however, the detection rates were similar to those reported in randomised studies conducted in primary care (0.5%– 3.0%).^8^ The results presented here are similar to those reported elsewhere,^5^^,^^11^^,^^21^^–^23 but highlight a new strategy. In a British study,^21^ new COPD cases were detected in 1.2% of participants sent a targeted COPD questionnaire, compared with 0.7% in those given the questionnaire when they next presented at their GP or practice nurse. A Dutch study sent all participants the Respiratory Health Screening Questionnaire: new COPD cases were diagnosed in 0.4% of patients in the patient-managed group and 1.3% of patients in the practice-managed group.^23^ Finally, an Australian study randomly allocated patients to a standard-care group or an intervention group that offered a consultation, including spirometry, with a practice nurse: new cases of COPD were detected in 0.2% of participants in the standard-care group, whereas the percentage was 2.5% for the intervention group.^22^ The results demonstrate that similar proportions of new COPD cases have been detected, despite differing healthcare systems and access to spirometry.

In the UK, nurses play a major role in primary care, which is not the case in France. Results of the study presented here show that interventions adapted to the French healthcare system detect COPD at levels that are comparable with those reported in other countries, and may suggest an innovative and complementary approach for other countries. In the DISCO study, patients were enrolled during only 4 months, compared with 5–12 months in other studies.^2^^,^^5^^,^^8^ The authors hypothesise that a delay in DISCO inclusion of >4 months would have increased the rate of new COPD cases.^2^^,^^5^^,^^8^

The DISCO study and the reported randomised studies all excluded patients who had already been diagnosed with COPD.^5^^,^^8^^,^^11^^,^^21^^–^23 Furthermore, all studies included patients aged >40 years, except for the British study, which had a lower age limit of 35 years. In contrast to the study presented here, the British and Australian studies included only patients with a history of smoking; in the DISCO study, only 47% of the patient participants were smokers. Duration of smoking exposure and number of pack–years were similar between the four groups;^27^^,^^28^ therefore smoking habits are unlikely to have biased the number of newly diagnosed COPD cases. The same interventions tested in DISCO but conducted only with smokers or ex-smokers might have revealed rates of new COPD cases that were closer to those of studies using this strategy.^8^^,^^22^

### Implications for practice and research

Organising the pathway to access spirometry and taking on the role of COPD coordinator can be done by medical assistants; when this study was undertaken, the medical assistant post had not been created in France, but it does exist in primary care in other countries. The study presented here shows that such organisation enables early detection of new cases of COPD. This is particularly useful for health systems in which spirometry is not performed in GP practices. Following the study presented here, a medico-economic evaluation of using a coordinator is being developed after DISCO.^29^

GPs allocated to a study intervention prescribed spirometry while none in the control group did so; this highlights the underuse of spirometry in primary care in real life. A further concern is that only 42% of prescribed spirometry was performed. The reasons for this require further study. This study was designed as an active case- finding study, adapted to French primary health care, to detect patients at risk of COPD. In a large proportion of patients identified as being at risk, spirometry was not performed, which highlights the critical need to not only identify patients at risk of COPD, but also to convince them to complete spirometry when it is prescribed.

Performing spirometry as soon as COPD is suspected would likely increase the number of new COPD cases detected. A recent pooled analysis of three Danish studies assessed the use of a mini- spirometry, then spirometry, to detect COPD in primary care;^30^^–^32 of the 6710 patients at risk, 17.7% were diagnosed with COPD, indicating that — as shown by the study presented here — early detection of COPD can be improved if use of spirometry in primary care practices is increased.

The search for risk factors and early symptoms of COPD has, therefore, made it possible to start a respiratory health pathway, not only for new COPD patients, but also for other respiratory pathologies diagnosed in the interventional groups.

Assessing COPD risk factors and symptoms in primary care allows GPs to detect COPD. GOLD questions alone can be a useful aid in identifying potential new COPD cases, and using a COPD coordinator to organise spirometry can increase the number of new cases of COPD detected. These interventions are relatively straightforward to implement in primary practice, particularly in countries such as France, where most GPs have not been trained to perform spirometry.

## Data

Study data available on reasonable request.
